# Genetic variation of long non-coding RNA TINCR contribute to the susceptibility and progression of colorectal cancer

**DOI:** 10.18632/oncotarget.16538

**Published:** 2017-03-24

**Authors:** Yongbin Zheng, Chao Yang, Shilun Tong, Yu Ding, Wenhong Deng, Dan Song, Kuang Xiao

**Affiliations:** ^1^ Department of Gastrointestinal Surgery, Renmin Hospital of Wuhan University, Wuhan, Hubei, PR China

**Keywords:** colorectal cancer, variation, genetic, TINCR, lncRNA

## Abstract

Colorectal cancer (CRC) accounts for the leading causes of cancer-related morbidity and mortality. However, a large part of heritable factors are warranted to be explored. Long non-coding RNAs (lncRNAs) serve critical roles in cancer development and progression. Herein, we explored effect of genetic variants of Tissue differentiation-inducing non-protein coding RNA (TINCR), a key lncRNA required for somatic tissue differentiation and tumor progression, on risk and progression of CRC. Three tagSNPs, including rs2288947, rs8105637, and rs12610531, were evaluated in in a two-stage, case-control study. Two SNPs, rs2288947 and rs8105637, were significantly associated with susceptibility of CRC in both stages. When pooled together, the allele G was significantly associated with 23% decreased risk of CRC (OR=0.77; 95% CI=0.67-0.88; P value = 1.2×10^−4^)for SNP rs2288947. While for SNP rs8105637, the allele A was significantly associated with 22% increased risk of CRC (OR=1.22; 95% CI=1.09-1.37; P value = 6.2×10^−4^). The two SNPs were also statistically associated with occurrence of lymph node metastasis of CRC. The carriers of allele G are less likely to get lymph node metastasis (OR=0.77; 95% CI=0.63-0.94; P value = 0.011) for rs2288947, and the carriers of allele A are more likely to get lymph node metastasis (OR=1.22; 95% CI=1.03-1.43; P value = 0.019) for rs8105637. These results suggest that lncRNA TINCR polymorphisms may be implicated in the development and progression of CRC.

## INTRODUCTION

Colorectal cancer (CRC) is one of the leading causes of cancer-related morbidity and mortality [1]. Except for advanced age, family history, male sex, and lifestyle factors which contribute to the increased risk of CRC, many genetic factors has been identified to be associated with susceptibility [1–6]. High-penetrance germline mutations, mismatch repair genes, together with identified loci from genome-wide association studies (GWAS), account for about 14% of the familial risk of CRC [7]. However, a large part of heritable factors are warranted to be explored [7, 8]. Further exploration of the interactive mechanism between genes and environment is helpful for specific diagnosis, screening, and personal treatment [9, 10].

With the innovations in sequencing technologies, long noncoding RNAs (lncRNAs) are being identified and characterized for serial steps of cancer development, including tumor initiation, growth, and metastasis [11–18]. Previously, we identified that the allele del of lncRNA GAS5 rs145204276 was significantly associated with 21% decreased risk of CRC [19]. Carriers of allele del are less likely to get lymph node metastasis, which should that GAS5 rs145204276 were significantly associated with the susceptibility and progression of CRC [19]. Here, we explored effect of genetic variants of another lncRNA on CRC risk in a case-control study, Tissue differentiation-inducing non-protein coding RNA (TINCR), a key lncRNA required for somatic tissue differentiation and tumor progression [20, 21]. Loss of TINCR expression promoted proliferation, metastasis through activating EpCAM cleavage in colorectal cancer [22].

## RESULTS

### Demographic characteristics

As shown in Table 1, the characteristics of the subjects were generally comparable in two stages, as no significant difference were detected for age group, gender, alcohol status and smoking status between CRC cases and healthy controls (all the P value > 0.05).

**Table 1:** The characteristics of the study population

Variables	Stage I			Stage II		
	Cases (n=600)	Controls (n=600)	P value	Cases (n=800)	Controls (n=800)	P value
Age group						
≥60	255 (42.5%)	264 (44.0%)	0.600	365 (45.6%)	362 (45.2%)	0.880
<60	345 (57.5%)	336 (56.0%)		435 (54.4%)	438 (54.8%)	
Gender						
Male	369 (61.5%)	372 (62.0%)	0.859	480 (60.0%)	468 (58.5%)	0.542
female	231 (38.5%)	228 (38.0%)		320 (40.0%)	332 (41.5%)	
Smoking status						
Smokers	186 (31.0%)	171 (28.5%)	0.344	232 (29.0%)	212 (26.5%)	0.264
Non-Smokers	414 (69.0%)	429 (71.5%)		568 (71.0%)	588 (73.5%)	
Alcohol status						
drinkers	201 (33.5%)	180 (30.0%)	0.193	280 (35.0%)	256 (32.0%)	0.204
Non-drinkers	399 (66.5%)	420 (70.0%)		520 (65.0%)	544 (68.0%)	
Tumor site						
Colon	340 (56.7%)			466 (58.2%)		
Rectum	260 (43.3%)			334 (41.8%)		
Lymph node metastasis						
No	390 (65.0%)			500 (62.5%)		
Yes	210 (35.0%)			300 (37.5%)		
Distant metastasis						
No	507 (84.5%)			688 (86.0%)		
Yes	93 (15.5%)			112 (14.0%)		

### Associations between TINCR polymorphisms and CRC susceptibility

**Figure 1:**
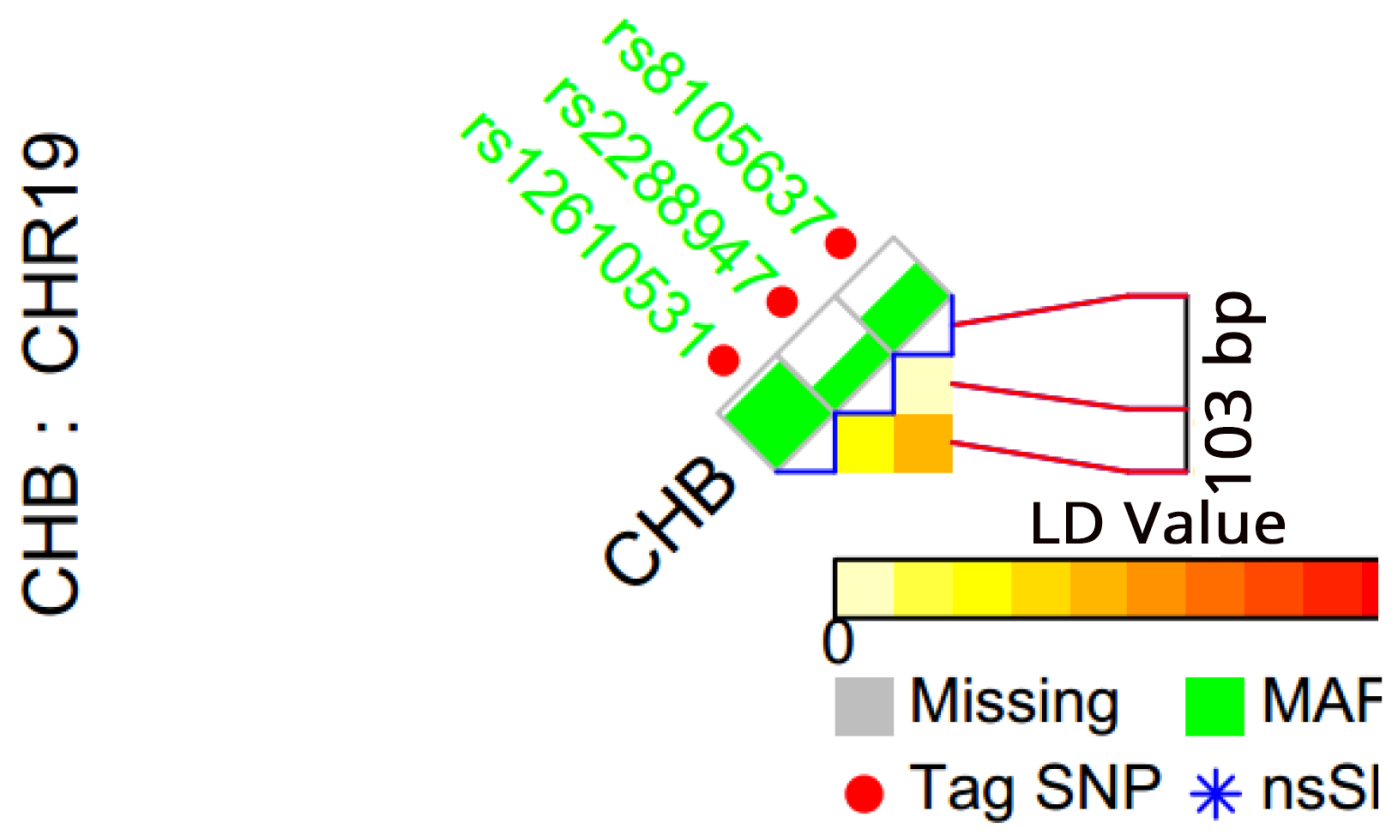
TagSNP selection for TINCR gene Red point represents a TagSNP, while gray square means “missing”, and green square means MAF.

Figure 1 shows the selection of tagSNPs for TINCR gene, including rs2288947, rs8105637, and rs12610531. The distribution of genotypes of all three tagSNPs in healthy controls in the two stage was in accordance with Hardy-Weinberg equilibrium (HWE, P > 0.05). As shown in Table 2, two SNPs, rs2288947 and rs8105637, were significantly associated with susceptibility of CRC in stage I (P=0.004 and 0.022, respectively). Thus, we replicated the associations of the two SNPs in an independent population (stage II, Table 3), which also presented statistically significant associations and same trend (P=0.007 and 0.009, respectively). When pooled together, the allele G was significantly associated with 23% decreased risk of CRC (OR=0.77; 95% CI=0.67-0.88; P value = 1.2×10^−4^) for SNP rs2288947. While for SNP rs8105637, the allele A was significantly associated with 22% increased risk of CRC (OR=1.22; 95% CI=1.09-1.37; P value = 6.2×10^−4^).

**Table 2:** Associations between TINCR gene polymorphisms and CRC susceptibility in stage I

Genotypes	Cases (n, %)	Controls (n, %)	OR (95% CI)^a^	P Value
rs2288947				
AA	384 (64.0%)	342 (57.0%)	1.00 (Reference)	
AG	195 (32.5%)	221 (36.8%)	0.78 (0.61-0.99)	**0.047**
GG	21 (3.5%)	37 (6.2%)	0.51 (0.46-1.03)	**0.010**
G vs A			0.75 (0.62-0.91)	**0.004**
rs8105637				
GG	281 (46.8%)	315 (52.5%)	1.00 (Reference)	
AG	264 (44.0%)	245 (40.8%)	1.21 (0.96-1.52)	0.106
AA	55 (9.2%)	40 (6.7%)	1.54 (1.01-2.35)	**0.045**
A vs G			1.22 (1.03-1.45)	**0.022**
rs12610531				
AA	186 (31.0%)	173 (28.8%)	1.00 (Reference)	
AG	310 (51.7%)	316 (52.7%)	0.91 (0.70-1.18)	0.489
GG	104 (17.3%)	111 (18.5%)	0.87 (0.62-1.22)	0.425
G vs A			0.93 (0.79-1.10)	0.411

^a^ adjusted by age, gender, alcohol and smoking status

**Table 3:** Associations between selected TINCR gene polymorphisms and CRC susceptibility in stage II

Genotypes	Cases (n, %)	Controls (n, %)	OR (95% CI)^a^	P Value
**rs2288947**				
Stage II				
AA	526 (65.8%)	480 (60.0%)	1.00 (Reference)	
AG	240 (30.0%)	270 (33.8%)	0.81 (0.66-1.00)	**0.049**
GG	34 (4.2%)	50 (6.2%)	0.62 (0.39-0.97)	**0.037**
G vs A			0.79 (0.69-0.94)	**0.007**
Pooled results				
AA	910 (65.0%)	822 (58.7%)	1.00 (Reference)	
AG	435 (31.1%)	491 (35.1%)	0.80 (0.68-0.94)	**0.006**
GG	55 (3.9%)	87 (6.2%)	0.57 (0.40-0.81)	**0.002**
G vs A			0.77 (0.67-0.88)	**1.2×10^−4^**
**rs8105637**				
Stage II				
GG	365 (45.6%)	412 (51.5%)	1.00 (Reference)	
AG	354 (44.3%)	328 (41.0%)	1.22 (0.99-1.50)	0.060
AA	81 (10.1%)	60 (7.5%)	1.52 (1.06-2.18)	**0.022**
A vs G			1.22 (1.05-1.42)	**0.009**
Pooled results				
GG	646 (46.1%)	727 (51.9%)	1.00 (Reference)	
AG	618 (44.2%)	573 (40.9%)	1.21 (1.04-1.42)	**0.014**
AA	136 (9.7%)	100 (7.1%)	1.53 (1.16-2.02)	**0.003**
A vs G			1.22 (1.09-1.37)	**6.2×10^−4^**

^a^ adjusted by age, gender, alcohol and smoking status

### Associations between TINCR polymorphisms and CRC susceptibility stratified by tumor site

The associations between rs2288947, rs8105637 and CRC susceptibility were analyzed by Tumor site (Table 4). In colon and rectum cancers, the trend was not materially changed.

**Table 4:** Associations between TINCR gene polymorphisms and CRC susceptibility stratified by tumor site

Genotypes	Colon cancer				Rectum cancer			
	Cases (n, %)	Controls (n, %)	OR (95% CI)^a^	P value	Cases (n, %)	Controls (n, %)	OR (95% CI)^a^	P value
**rs2288947**								
AA	540 (67.0%)	822 (58.7%)	1.00 (Reference)		370 (65.0%)	822 (58.7%)	1.00 (Reference)	
AG	242 (30.0%)	491 (35.1%)	0.75 (0.62-0.91)	**0.003**	193 (31.1%)	491 (35.1%)	0.87 (0.71-1.07)	0.199
GG	24 (3.0%)	87 (6.2%)	0.42 (0.27-0.66)	**1.7×10^−4^**	31 (3.9%)	87 (6.2%)	0.79 (0.52-1.21)	0.284
G vs A			0.70 (0.60-0.82)	**1.3×10^−5^**			0.88 (1.03-0.74)	0.117
**rs8105637**								
GG	370 (45.9%)	727 (51.9%)	1.00 (Reference)		276 (46.5%)	727 (51.9%)	1.00 (Reference)	
AG	354 (43.9%)	573 (40.9%)	1.21 (1.01-1.46)	**0.037**	264 (44.4%)	573 (40.9%)	1.21 (0.99-1.48)	0.059
AA	82 (10.2%)	100 (7.1%)	1.61 (1.17-2.21)	**0.003**	54 (9.1%)	100 (7.1%)	1.53 (0.99-2.03)	0.053
A vs G			1.24 (1.09-1.42)	**0.001**			1.19 °(1.03-1.38)	**0.018**

^a^ adjusted by age, gender, alcohol and smoking status

### Associations between TINCR polymorphisms and lymph node metastasis and distant metastasis of CRC

We also investigated the associations between rs2288947, rs8105637 and Lymph node metastasis and Distant metastasis of CRC. As shown in Table 5, the carriers of allele G are less likely to get lymph node metastasis (OR=0.77; 95% CI=0.63-0.94; P value = 0.011) for rs2288947, and the carriers of allele A are more likely to get lymph node metastasis (OR=1.22; 95% CI=1.03-1.43; P value = 0.019) for rs8105637. Due to the limited sample size and statistical power, the associations with distant metastasis of CRC were not significant (P>0.05).

**Table 5:** Associations between TINCR gene polymorphisms and Lymph node metastasis and Distant metastasis of CRC

Genotypes	Lymph node metastasis				Distant metastasis			
	Event (n, %)	No event (n, %)	OR (95% CI)^a^	P _trend_	Event (n, %)	No event (n, %)	OR (95% CI)^a^	P_trend_
**rs2288947**								
AA	352 (69.0%)	552 (62.0%)	1.00 (Reference)		137 (66.8%)	773 (64.7%)	1.00 (Reference)	
AG	140 (27.5%)	298 (33.5%)	0.74 (0.58-0.94)		61 (31.3%)	374 (31.3%)	0.92 (0.66-1.27)	
GG	18 (3.5%)	40 (4.5%)	0.70 (0.40-1.25)		7 (3.4%)	48 (4.0%)	0.82 (0.37-1.85)	
G vs A			0.77 (0.63-0.94)	**0.011**			0.91 (0.70-1.20)	0.517
**rs8105637**								
GG	215 (42.1%)	431 (48.4%)	1.00 (Reference)		89 (43.4%)	557 (46.6%)	1.00 (Reference)	
AG	238 (46.7%)	380 (42.7%)	1.25 (1.00-1.58)		93 (45.4%)	525 (43.9%)	1.11 (0.81-1.52)	
AA	57 (11.2%)	79 (8.9%)	1.45 (0.99-2.11)		23 (11.2%)	113 (9.5%)	1.27 (0.77-2.10)	
A vs G			1.22 (1.03-1.43)	**0.019**			1.12 (0.90-1.40)	0.319

^a^ adjusted by age, gender, alcohol and smoking status

## DISCUSSION

The current study systematically explored the potential associations between three tagSNPs of lncRNA TINCR, including rs2288947, rs8105637, and rs12610531, and risk and progression of CRC in in a two-stage, case-control study in Chinese population. To be best of our knowledge, this should be the first study which aims to evaluated the associations between genetic variation of lncRNA TINCR and susceptibility and progression of CRC.

Recent studies have elucidated the important role and mechanism of lncRNAs in cancer development and progression, although the specific functions of most lncRNAs remain unknown [13, 14, 16–18, 23–25]. The most focused lncRNA, HOTAIR which cooperate with Polycomb complex PRC2 and reprogram chromatin organization, could promote cancer metastasis in colorectal cancer [26]. Its genetic variations have been confirmed to be associated with susceptibility of ovarian cancer, cervical cancer, breast cancer, and gastric cancer [27–32]. Very recently, Ma et al [33] reported tagSNPs of lncRNA TINCR could affect the genetic susceptibility to gastric cancer in a Chinese population. Consistent with our results, they found, the variant AG, GG, and GG+AG genotypes and G allele of rs2288947 were correlated with a remarkably reduced risk of GC (P= 0.026, 0.026, 0.008 and 0.037 respectively), compared with the AA genotype and A allele [33]. Different with our results, rs8105637 was not associated with GC risk in the report of Ma et al [33]. Using HaploReg v4.1 [34], we found rs2288947 could alter 5 motifs, including CTCF_disc9, Nanog_disc3, Rad21_disc10, SMC3_disc3, and SP1_disc3. While rs8105637 could alter expression of Pitx2, TCF12. These motifs have been confirmed to be associated with carcinogenesis and metastasis [35–40].

In the stratified analyses, we observed difference in the association between rs2288947 genotype and CRC risk according to tumor site. The association was more significant for colon cancer while not significant for rectal cancer, although the exact mechanisms for these differences are currently unclear. We also didn't detected significant association between lncRNA TINCR rs2288947, rs8105637 and distant metastasis of CRC. They might be caused by to the limited sample size of the event cases and the insufficient statistical power.

Our study has several strengths. First, the implement of the two-stage, case-control study design, which is suggested for genetic association studies [41, 42]. Second, we have sufficient statistic power to detect such associations. Using QUANTO software (http://biostats.usc.edu/Quanto.html), we found that the statistic power for the log additive model of rs2288947 was 98%, and 92% for that of rs8105637. There are also limitations in the current study. Such as the lack of independent replication with different ethnic background, and mechanism research. Further investigations are required to gain insight into the mechanisms by which TINCR regulates the occurrence progress of CRC.

Taken together, this is the first study demonstrating the potential associations between genetic variation of lncRNA TINCR with susceptibility and progression of CRC in Chinese population. Our results firstly indicate that SNP rs2288947 and rs8105637 may act as independent biomarkers associated with occurrence and progression of CRC. This study provided valuable clues for better understanding the underlying contribution of genetic variation of lncRNA TINCR to carcinogenesis of CRC. Future functional studies should be conducted to further explore the role of lncRNA TINCR in the development and progression of CRC basing on the epidemiological findings.

## PATIENTS AND METHODS

### Study subjects

In this two-stage, cases-control study, we totally recruited 1400 CRC cases and 1400 healthy controls between 2010 and 2015, which were matched by age group, gender, alcohol and smoking status. We have described these in a previous study which evaluated the functional of LncRNA GAS5 in development and progression of CRC [19]. Five milliliter peripheral blood was collected from all subjects, and demographic information were face to face interviewed by the project staff. The study was approved by appropriate Research Ethics Committee (REC) of Renmin Hospital of Wuhan University, and written informed consent was obtained from all participants.

### TagSNP selection, DNA extraction and genotyping

TagSNP selection was conducted using SNPinfo (https://snpinfo.niehs.nih.gov/). Qiagen genomic DNA purification kit were used for extraction of the genomic DNA from blood samples. Genotyping was performed using the TaqMan allelic discrimination assay on the ABI PRISM 7900HT Sequence Detection System. The genotyping results were determined by using the SDS 2.3 Allelic Discrimination Software (Applied

Biosystems, Carlsbad, CA). Quality control was conducted by direct sequencing 5% duplicate samples in blind, with a concordance rate of 100%. Furthermore, a 5% random selected sample was replicated in duplicate by different persons, and the concordance rate was 100%.

### Statistical analysis

Unconditional Logistic regression model was used to calculate the Odds ratios (ORs) and 95% confidence intervals (95% CIs) for the associations between TINCRpolymorphisms and risk of CRC and its Lymph node metastasis and Distant metastasis, adjusted for age group, gender, alcohol and smoking status. Hardy-Weinberg equilibrium was tested for with a goodness of fit χ2 test with one degree of freedom to compare the observed genotype frequencies among the subjects with the expected genotype frequencies. All statistics were performed using SPSS software 19.0 (SPSS Inc., Chicago, IL, USA), and P values were two sided with the statistical significance criteria of P < 0.05 all through the study.
